# Uncovering the Genetic Structure of European Anchovy Populations in Central and Western Mediterranean

**DOI:** 10.1002/ece3.72441

**Published:** 2025-11-18

**Authors:** Damianos Alexandridis, Tereza Manousaki, Aglaia Antoniou, Jon Kristoffersen, Charis Apostolidis, Rita Cannas, Maria Teresa Spedicato, Alessia Cariani, Jose María Bellido, Antonios Magoulas, Francisco Ramírez, Elena Lloret‐Lloret, Marta Albo‐Puigserver, Marta Coll, Costas S. Tsigenopoulos

**Affiliations:** ^1^ Institute of Marine Biology, Biotechnology and Aquaculture (IMBBC) Hellenic Centre for Marine Research (HCMR) Heraklion Greece; ^2^ Department of Biology University of Crete Heraklion Greece; ^3^ Institute of Marine Biological Resources and Inland Waters (IMBRIW) Hellenic Centre for Marine Research (HCMR) Heraklion Greece; ^4^ Department of Life and Environmental Sciences University of Cagliari Cagliari Italy; ^5^ CoNISMa Rome Italy; ^6^ COISPA Technology and Research Bari Italy; ^7^ University of Bologna Bologna Italy; ^8^ Instituto Español de Oceanografía (IEO‐CSIC), Centro Oceanográfico de Murcia Murcia Spain; ^9^ Institute of Marine Science (ICM‐CSIC), Passeig Marítim de la Barceloneta Barcelona Spain; ^10^ Research Institute of the University of Bucharest (ICUB) University of Bucharest Bucharest Romania; ^11^ Instituto Español de Oceanografía (IEO‐CSIC), Centro Oceanográfico de Baleares Barcelona Spain

**Keywords:** ddRAD, *Engraulis encrasicolus*, genome assembly, population genomics, seascape genomics, SNP

## Abstract

Among small pelagic fishes, the European anchovy ( *Engraulis encrasicolus* ) is a key species within the marine ecosystem of the Mediterranean Sea, exhibiting significant population structure across its geographic range. This study applied state‐of‐the‐art genomic analyses, utilizing 9497 genome‐wide SNPs derived from ddRAD sequencing, to investigate population structure, genetic diversity, and genotype‐environment associations in anchovy samples collected across 12 locations in the Western and Central Mediterranean as well as nearby Atlantic regions. In order to increase the number of polymorphic loci identified, we first assembled a draft reference genome for the species (1.69 Gb and 79.8% BUSCO completeness), which proved to be a powerful tool for downstream analyses. Two main genetic clusters were delineated: one comprising individuals from the Atlantic and Alboran Sea and the other including those from the Northwestern (particularly Balearic Sea and Gulf of Lion) and Central Mediterranean (*F*_ST_ = 0.09), indicating the Almeria–Oran front as a potential dispersal barrier for gene flow in the species. Seascape genomic analyses identified statistically significant associations between SNPs and environmental variables such as temperature and nutrient availability. Overall, our research highlighted the genetic relationships among anchovies in the studied area, providing essential insights needed for identifying distinct management units and developing conservation strategies, while emphasizing the need to address genetic and environmental dynamics in light of climate change, which may threaten the stability and resilience of anchovy populations and their habitats.

## Graphical abstract

This study used advanced genomic techniques to analyze population structure, genetic diversity, and environmental associations in European anchovies across the Western and Central Mediterranean and nearby Atlantic regions. Two main genetic clusters were identified, separated by the Almeria–Oran front, with significant associations found between genetic variation and environmental factors like temperature, salinity, and nutrients. These findings provide crucial insights for the conservation and management of anchovy populations, especially under the pressures of climate change.

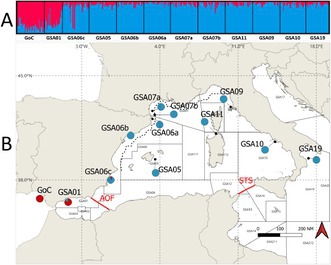

## 1 Introduction

The alarming decline in biodiversity over recent decades may significantly affect ecosystem functioning, with profound consequences for human well‐being (Cardinale et al. 2012; O'Hara et al. 2021). The Mediterranean Sea, a global hotspot for marine biodiversity, is among the most threatened regions of the planet (Ramírez et al. 2021; Coll et al. 2015). Human‐induced pressures such as pollution, overfishing, and climate change are the primary threats to the Mediterranean basin (Halpern et al. 2008; Coll et al. 2010; Costello et al. 2010). Direct consequences of these drastic alterations include declines in commercially important small pelagic fish populations (Coll et al. 2019; Piroddi et al. 2017; Van Beveren et al. 2016) in the Mediterranean, which still constitutes an important fishing ground (Ramírez et al. 2018). However, signs of reversing the continuous decline of certain fisheries resources were observed in the last decade with the percentage of overfished stocks following a remarkable decreasing trend, an improvement consistent with a continuous reduction in fishing pressure in the same period (FAO 2023).

The appropriate assessment and management of fish stocks is essential in addressing these challenges. This can be achieved by first identifying the population structure of particular species and describing their main characteristics in terms of genetic diversity, adaptation, and connectivity (Andersson et al. 2024). Genomics can provide deeper insights into these characteristics and thus foster more tailored fisheries management actions (Andersson et al. 2024). Furthermore, by incorporating environmental data, it is possible to evaluate how environmental factors influence selective and neutral genomic diversity in natural populations, as studied in the field of seascape genomics (Riginos et al. 2016). Identifying such locus‐by‐environment associations provides insights into the way the environment interacts with natural populations, which can inform conservation strategies by guiding the prioritization of efforts—for example, by identifying habitats that maintain adaptive genetic diversity or by defining spatial management measures, such as protected areas or fishing regulations (quotas and exclusion zones)—especially in the current context of global environmental changes driven by climate and human activity (Riginos et al. 2016; Rieder et al. 2025).

The European anchovy ( *Engraulis encrasicolus* L. 1758), a key species in the Mediterranean ecosystem, is a small pelagic, schooling fish commonly inhabiting both marine and brackish waters (Checkley et al. 2017). It is widely distributed across the northeastern and central Atlantic, the Mediterranean, the Black and Azov seas, as well as along the coasts of West to South Africa (Whitehead 1984; Whitehead et al. 1988). This short‐lived, planktivorous species undergoes multiple spawning events from spring to autumn in the Mediterranean, with peaks in June and July (Palomera et al. 2007; Somarakis et al. 2004). Spawning activity is strongly influenced by temperature, though other environmental factors may also drive variations in peak spawning periods (Millân 1999; Motos et al. 1996; Palomera 1992; Palomera et al. 2007).

Small pelagic fish, such as the European anchovy, are crucial to coastal ecosystems, acting as key intermediaries in the transfer of energy from lower to higher trophic levels (Cury et al. 2000). These species often form narrow “wasp‐waist” ecosystems, characterized by low species diversity but high abundance (Bakun 2006; Jordán et al. 2005). Shifts in their relative abundance can have significant ecological consequences, inducing both bottom‐up effects—where changes in small pelagic fish abundance, driven by environmental factors, impact their predators—and top‐down effects through predation pressures (Schwartzlose et al. 1999; Cury et al. 2011).

In addition to the anchovy's crucial role in marine ecosystems, its cultural and economic importance in supporting fisheries cannot be overlooked. In the Western Mediterranean basin, local pelagic fisheries highlight not only their contribution to the local economy but its cultural significance in the region (Pertierra and Lleonart 1996). In 2020 and 2021, the European anchovy was one of the main landed species in the Western and Central Mediterranean accounting for 26,623 t (13%) and 8263 t (4.7%), respectively (FAO 2023), while in the Adriatic Sea, the anchovy is one of the most important stocks (2024 catches: 26,769 tons; https://www.fao.org/gfcm/data/safs) managed by a Multi Annual Management Plan (Recommendation GFCM/44/2021/20) and with recent signs of improvement in its status (GFCM 2024 data). Finally, the European anchovy is also known for its high nutritional profile, since essential fatty acids like Ω‐3 (DHA, EPA) and Ω‐6 (ARA) are found in its flesh (Zlatanos and Laskaridis 2007).

In recent decades, European anchovy populations in the Mediterranean have shown significant changes in biological traits such as body condition, length‐at‐age, and length at maturity, alongside fluctuations and overall declines in abundance and biomass (Brosset et al. 2017; Van Beveren et al. 2016; Albo‐Puigserver et al. 2021; Pennino et al. 2020). Albo‐Puigserver et al. (2021) described long‐term patterns in these traits for anchovy and sardine ( *Sardina pilchardus* ) that differ between northern and southern areas of the Western Mediterranean, with more pronounced declines observed in northern regions. Notably, they reported latitudinal differences in gonadosomatic index and body condition at seasonal and interannual levels, further emphasizing the complexity of environmental drivers affecting small pelagic fish populations. For example, anchovies in the Alboran Sea exhibit higher growth rates than those in the Ebro Delta, highlighting the importance of local environmental conditions (Alemany and Alvarez 1993). While shifts in plankton composition, fishing pressure, and environmental changes have been proposed as key drivers of anchovy decline (Pennino et al. 2020; Menu et al. 2022), the observed regional differences suggest that genetic factors may be involved. Understanding how environmental factors influence anchovy genomic diversity at both large and local scales is therefore essential for effective fisheries management and conservation.

To gain deeper insight into these environmental influences, it is essential to identify the key physical and chemical factors affecting small pelagic fish. Physical variables, such as temperature (Palomera 1992), salinity (Fernández‐Corredor et al. 2021), bathymetry (Fernández‐Corredor et al. 2021), and hydrodynamics (Falcini et al. 2015), as well as chemical variables, including nitrate (Checkley et al. 2017), silicate (Silva et al. 2014), phosphate (Silva et al. 2014), and oxygen (Bertrand et al. 2011), have been reported to influence anchovy populations.

The European anchovy's populations and their evolutionary history have been studied by means of morphological (Spanakis et al. 1989; Tudela 1999), acoustic (Chashchin 1996), and molecular data (e.g., Bembo et al. 1996; Borsa 2002; Magoulas et al. 1996; Spanakis et al. 1989), revealing remarkable population structure throughout its distribution area (e.g., Grant 2005). In particular, the Western Mediterranean anchovy populations (except the Alboran Sea), have been found genetically closer to those of the North Sea, English Channel, and Bay of Biscay than to those of the East Atlantic coast (Gulf of Cadiz, Alboran Sea, Portugal, Canary Islands) (Zarraonaindia et al. 2012 using SNPs). This pattern has been corroborated in other studies as well (Magoulas et al. 1996, 2006 with RFLPs; Viñas et al. 2013 with mtDNA control region; Catanese et al. 2020 and Zarraonaindia et al. 2009 with microsatellites).

Within the Mediterranean basin, there is a pronounced population structure, which results from recent and historical factors (Grant 2005; Magoulas et al. 2006). In the basin, the population structure varies between its eastern and western parts and might be attributed to sharp water salinity changes (Magoulas et al. 1996) in conjunction with physical barriers that limit gene flow within the basin. In particular, studies using morphometric and genetic data, in the Adriatic, Ionian, and Aegean Seas, have shown population substructure in the Eastern Mediterranean (Spanakis et al. 1989). On the other hand, within the Western Mediterranean only the Alboran population is found to be genetically distinct from those from the Balearic Sea, Gulf of Lion, Ligurian, and Tyrrhenian Seas (Sanz et al. 2008; Tudela 1999; Viñas et al. 2013). This is probably due to the Alboran Sea's oceanographic characteristics, which constitute a transition zone between the Atlantic Ocean and the Mediterranean Sea (Patarnello et al. 2007). Its waters are more similar, in terms of chemical components, to those of the North East Atlantic than to those of the Western Mediterranean, shaping the Almeria–Oran Front (AOF; Tintore et al. 1988) with its particular oceanographic conditions acting as a barrier to dispersal for many marine organisms as well as for the European anchovy (see for example Bouchenak‐Khelladi et al. 2008).

While previous studies have extensively characterized the population structure of the European anchovy throughout its range, our research builds on this foundation focusing on a local scale with genome‐wide markers and employing a seascape genomics approach. Specifically, we integrate novel genetic markers (SNPs) obtained through ddRAD sequencing, increase sampling effort at a local scale, expand sampling locations to further resolve fine‐scale population structure, and incorporate environmental data to test whether environmental factors shape genetic differentiation in anchovy populations. To achieve this, we first generated a draft whole genome assembly of the species, which was then used as a reference. This allowed us to delineate a high number of reliably inferred loci from the ddRAD dataset, resulting in high resolution in our analyses. A seascape genomic approach was followed to assess how the genomic variability is influenced by certain environmental variables. Our large genomic dataset allowed us to conduct an in‐depth population genomic analysis, establishing the genetic relationships of anchovy samples across various geographical sites in the Western and Central Mediterranean, as well as in adjacent Atlantic regions, rendering our research crucial for fishery and conservation strategies.

## 2 Materials and Methods

**FIGURE 1:**
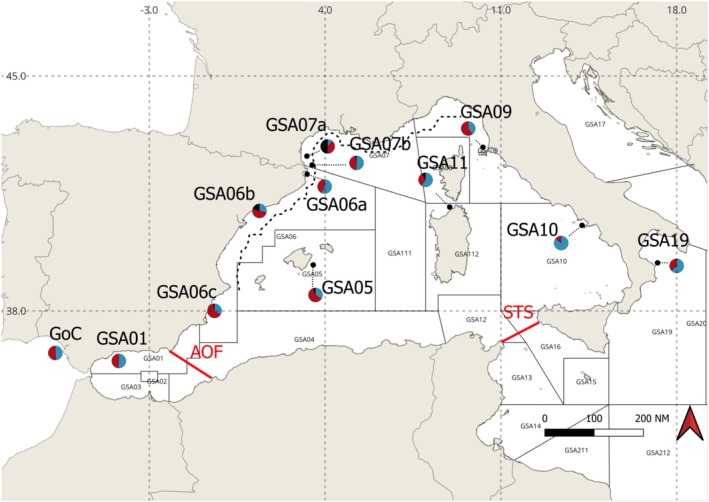
Map of the 12 sampling sites under study. FAO area 37. Colors in pies indicate the proportion of males (blue), females (red), and immatures (black) captured. GoC = Gulf of Cadiz, GSA01 = Northern Alboran Sea, GSA05 = Balearic Islands, GSA06 = Northern Spain, GSA07 = Gulf of Lion, GSA09 = Ligurian Sea, GSA10 = Tyrrhenian Sea, GSA11 = Sardinia, GSA19 = Western Ionian Sea. The Almeria–Oran Front and the Siculo–Tunisian Strait are indicated by red lines, while the Ligurian Current is shown with a black dashed line.

Anchovy individuals were collected from 12 different locations (Figure 1) in the Western and Central Mediterranean, as well as the adjacent Atlantic waters, during scientific surveys (Mediterranean International bottom Trawl Survey, MEDITS, Spedicato et al. 2019; and Mediterranean International Acoustic Survey, MEDIAS, Giannoulaki et al. 2021) or commercial hauls in the period from November 2017 to June 2018. A total of 408 anchovies (197 males, 176 females, 35 immatures) were sampled (Figure 1 and Table S1) from several Geographical Sub‐Areas [GSAs, as defined by the General Fisheries Commission for the Mediterranean (GFCM)]. Fin‐clips were taken from a sub‐sample of dead fish collected during the routine campaigns of the mentioned surveys. All partners involved in the sampling activities stored the fin‐clips individually in tubes with non‐denatured ethanol 96%. After completion of the sampling, tissues were shipped and collected by ICM/CSIC. These samples were used to construct double‐digest random amplified DNA (ddRAD) libraries following the protocol described in Kess et al. (2016). Additionally, one female anchovy specimen collected from the Northern Euboean Gulf (Greece) in May 2023 was used for constructing a European anchovy draft genome.

Sampling was conducted exclusively in offshore marine environments across the Mediterranean Sea. No samples were collected in inshore, estuarine, or brackish habitats. Based on current ecological and taxonomic knowledge, *Engraulis encrasicolus* is the only Engraulis species known to inhabit offshore marine waters in the region. Therefore, all sampled individuals in this study are considered to belong to *E. encrasicolus* .

### 2.1 Draft Genome Assembly of the European Anchovy: DNA Extraction, Library Preparation, Nanopore Sequencing and Assembly

DNA was extracted from a single anchovy specimen using the standard salt‐based protocol of Miller et al. (1988). For the genome assembly, since the extracted DNA was moderately fragmented, low molecular weight DNA was depleted with a size selection protocol using PEG 8000 (Polyethylene Glycol) and NaCl (Tyson 2020). Library construction and sequencing were performed using a total of three Oxford Nanopore flow cells. Two libraries were constructed using the SQK‐LSK110 and SQK‐LSK114 sequencing kits and sequenced on an R9.4.1 flow cell and two MinION R10.4.1 flow cells, respectively. Guppy v6.5.7 and Dorado v0.4.1 were used for basecalling. Flye v2.9 (Kolmogorov et al. 2019) was used for the *de novo* assembly with the following parameters: ‐‐genome‐size 1.5 g to provide the anchovy's estimated genome size of 1.5 Gb and ‐‐scaffold to enable scaffolding. The generated genome assembly was first polished by Flye and then by Racon v1.4.20 (Vaser et al. 2017). The resulting assembly was evaluated for gene content with BUSCO (Benchmarking Universal Single‐Copy Orthologs, Manni et al. 2021) using the *actinopterygii_odb10* lineage dataset. Finally, BWA v0.7.15 (Li and Durbin 2009) was used to index the reference genome.

### 2.2 ddRAD Data Generation and Bioinformatic Analysis

Genomic DNA was extracted from tissue samples using the E.Z.N.A. Mollusk DNA kit (Omega Biotek). The isolated DNA was quantified using the Qubit dsDNA BR assay (Thermo Fisher Scientific). Genomic libraries were prepared using the ddRAD sequencing approach developed by Kess et al. (2016) with two six‐cutter enzymes, *PstI* (CTGCA|G) and *BglII* (A|GATCT). Sequenced reads were analyzed using the STACKS v2.64 pipeline (Catchen et al. 2013), to quality control the reads (applying the *‐c, ‐q and ‐r* parameters in https://process_radtags.pl), identify the genomic loci sequenced (using the following parameters: ‐R 0.8, –max‐obs‐het 0.5, –min‐maf 0.05, −write‐single‐snp in *populations*) and genotype each individual, following a reference‐based approach with the generated genome assembly used as the reference. Finally, SNPs in linkage disequilibrium (*r* ^2^ > 0.5) were pruned using Plink v1.9 (Purcell et al. 2007) and a missing data threshold of 20% was applied in the dataset (at the level of individuals and genetic loci). The paired‐end reads of each individual were mapped to the generated genome with BWAv0.7.15. The generated sam files were converted to bam files with SAMtools v1.9 (Li et al. 2009).

### 2.3 Population Genomic Analyses

To assess the population structure of the species, STRUCTURE v2.3.4 (Pritchard et al. 2000) and discriminant analysis of principal components (DAPC, Jombart 2008) were applied. STRUCTURE was performed under the correlated allele frequency model, allowing for admixture. Individuals with a membership coefficient above 0.9 were assigned to clusters, while those below this threshold were considered to have mixed ancestry (admixed). Each run had a burn‐in length of 250,000 and 500,000 MCMC (Markov Chain Monte Carlo) iterations. The number of predetermined genetic clusters (K) tested ranged from 1 to 7, with 10 replicates per K. Structure threader (Pina‐Martins et al. 2017) was used to parallelize and automate the runs of STRUCTURE. Structure harvester was utilized for the implementation of the Evanno method (Evanno et al. 2005) and the provision of Q‐matrices for CLUMPP, a cluster matching and permutation program, and DISTRUCT, a visualization program used to graphically display genetic clustering results (Earl and vonHoldt 2012; Jakobsson and Rosenberg 2007; Rosenberg 2004).

DAPC was performed with the adegenet v2.1.1 R package (Jombart 2008) to infer population subdivision independently of population genetics models. Before running the Discriminant Analysis (DA), the number of principal components achieving the highest mean success was identified using a stratified cross‐validation with the function *xvalDapx* of the adegenet v2.0.0 R package (Jombart 2008). The DA was then run on the retained principal components using the *dapc* function. Finally, after selecting the best number of eigenvalues for the DA analysis, the DAPC results (DAPC scatterplots) were visualized graphically with the *scatter* function of the ade4 R package (Dray and Dufour 2007). To determine the number of expected genetic clusters (K) present in the dataset, without any a priori population definition, the *find.clusters* function was used to run a series of K‐means clusters of the individuals across a range of *K* = 1–7. We identified the best supported number of clusters through comparison of the Bayesian Information Criterion (BIC) for the different values of *K*.

Estimates of expected and observed heterozygosity between the 12 sampling sites and the identified clusters were estimated using the hierfstat R package (Goudet 2005). Genetic differentiation among sites and clusters was assessed using Wright's fixation index (F_ST_), with statistical significance evaluated through 10,000 iterations.

### 2.4 Seascape Genomic Analyses

**FIGURE 2:**
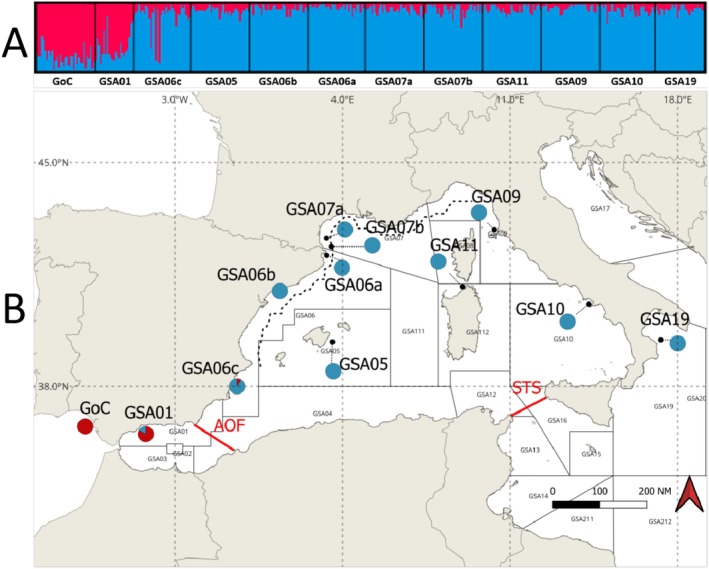
Population structure of European anchovy in the Eastern Atlantic and Western and Central Mediterranean Sea as inferred by (A) STRUCTURE for *K* = 2 with the bar plot indicating individual q‐values with all SNPs. (B) DAPC with *K* = 2 where the proportions of individuals assigned with posterior probability equal to one to the two clusters, that is, ATL in red and MED in blue, are depicted per sampling site. The Almeria–Oran Front and the Siculo–Tunisian Strait are indicated by red lines, while the Ligurian Current is shown with a black dashed line.

Three types of genomic datasets were analyzed to determine whether variations in population structure were influenced by certain environmental variables. The first dataset included all sampling locations (named as “all samples”). The other two datasets consisted of the two genetic clusters (“MED” and “ATL”) detected by the population genomic analyses (e.g., Figure 2A).

### 2.5 Environmental Variables

Environmental variables were initially selected to include all previously reported variables and their trends with significant impact on anchovies, such as temperature (Basilone et al. 2004; Mutalipassi et al. 2024), food availability (Schwartzlose et al. 1999; including nitrate (Checkley et al. 2017), silicate (Silva et al. 2014) and phosphate (Silva et al. 2014)), salinity (Fernández‐Corredor et al. 2021), bathymetry (Fernández‐Corredor et al. 2021), and oxygen (Bertrand et al. 2011), as well as variables related to the biology of the species whose impact has never previously been assessed with respect to the species' evolutionary history, as well as climate variability, as the dominant processes affecting small pelagic fish. A total of 32 environmental variables were included from four sources (Table S2): (a) 18 geophysical and climatic data layers from MARSPEC (Sbrocco and Barber 2013), (b) 8 climatic, biological, and geophysical environmental layers from Global Marine Environment Datasets (GMED, Basher et al. 2018), (c) 5 variables on spatio‐temporal trends in sea temperature as a proxy to optimal environmental conditions for anchovy biomass and spawning (based on Ramírez et al. 2018, 2021) and (d) EMODnet data (www.emodnet‐seabedhabitats.eu; see Table S1 for more details). Raster values from all layers at the sampling sites were collected with the Point Sampling Tool plugin of QGIS v. 3.34.3 (QGIS.org, 2021, QGIS Geographic Information System, QGIS Association http://www.qgis.org).

### 2.6 Outlier Detection

Following the methods outlined by Antoniou et al. (2023), in this study three approaches were utilized for outlier detection. These included the univariate genotype‐environment association (GEA) method gINLAnd (Guillot et al. 2014); a PCA‐based approach using the PCadapt R package (Luu et al. 2017) with Benjamini‐Hochberg correction; and a redundancy analysis (RDA) using the vegan R package (Oksanen et al. 2012).

SNPs identified by all three methods (gINLAnd, PCadapt, RDA) were considered outliers, whereas the putative neutral SNP dataset included SNPs that were not identified by any of the methods. All computationally intensive analyses of the study were conducted at the IMBBC HPC facility (Zafeiropoulos et al. 2021).

## 3 Results

### 3.1 Draft Genome Assembly

Nanopore sequencing yielded around 14.7 GB of data in total. Nearly 13.45 GB of data were successfully base called, of which 94.4% was simplex and 5.6% was duplex. The base called data account for 3,668,883 reads with N50 of 5,923 bp.

Flye pipeline assembled a genome of total length 1.69 Gb, which contained 76,551 contigs (contig N50: 57,292 bp) and 48 scaffolds (scaffold N50: 422,847 bp) with 9× coverage and N50: 57,970 bp in total. BUSCO assessment found 2905 (79.8%) completed, 257 fragmented (7.1%), and 478 (13.1%) missing BUSCOs (Benchmarking Universal Single‐Copy Orthologs).

### 3.2 ddRAD Data Analysis

Illumina sequencing yielded 983,601,116 raw reads, 2.5% of which were discarded from the *process_radtags* quality control. After alignment against the genome, more than 98% of the retained reads per individual were mapped to the genome. The *gstacks* program generated a catalog of 410,701 loci with a mean insert length of 192.2 bp (sd: 63.5) and effective coverage per individual of 28.7× (stdev = 7.8×). The *populations* program removed 396,901 out of 410,701 loci that did not pass sample or population constraints. From the remaining 13,800 loci, 9645 variant sites occurred with a mean genotyped sites per locus of 229.25 bp (stderr 0.59). In the *populations* output file (VCF format), additional filtering was applied in terms of data missingness and genetic association between SNPs. The final VCF file consists of 398 individuals genotyped at 9497 SNPs.

### 3.3 Population Structure and Genetic Differentiation

Bayesian clustering analysis with STRUCTURE was conducted on the “all samples” dataset, and the optimal K, as estimated by the Evanno method, was *K* = 2 (Figure 2A). The first cluster (“ATL”) consists of individuals with *Q* value > 0.9 (individuals in red in Figure 2A), mainly from GoC (Gulf of Cadiz) and GSA01 (Alboran Sea), while the rest of the individuals belong to the second cluster (“MED”). Moreover, all localities seemed to have some admixed individuals (Figure 2A). The GSA01, GoC and GSA06c sampling sites had the most admixed individuals with 100%, 80% and 54%, respectively, while the GSA06b and GSA06a had the fewest admixed individuals with just 11%.

DAPC analysis of the “all samples” dataset resulted in defining the same two clusters as in STRUCTURE (*ATL* and *MED* clusters) (Figure 2B). All individuals were assigned to two clusters, but no admixed samples were found (using all or neutral loci; Figure S1), in contrast to STRUCTURE's results. Under a hierarchical scheme, we examined whether there was further substructure within the detected clusters. This was only possible for the *MED* cluster since the *ATL* cluster consisted of a low number of individuals. According to the results, no substructuring occurred within the *MED* cluster.

The genetic pattern observed in Figure 2A is also supported by the *F*_ST_ index (Table S3). The GoC and GSA01 anchovies, which make up the *ATL* cluster, share high *F*_ST_ values with all the other Mediterranean anchovies and at the same time they have low *F*_ST_ (0.0043) among them (Table S3). Low *F*_ST_ values, but only a few of them significant, were evident for the *MED* group. In addition, similar levels of expected and observed heterozygosity were estimated across all sites (Ho = 0.174–0.188 and He = 0.209–0.243). Finally, the *F*_ST_ between the *ATL* and *MED* clusters was found to be 0.097 (*p* < 0.05) with similar levels of expected and observed heterozygosity (*ATL*: 0.180 Ho and 0.217 He, *MED*: 0.186 Ho and 0.242 He).

### 3.4 Seascape Genomic Analyses

**FIGURE 3:**
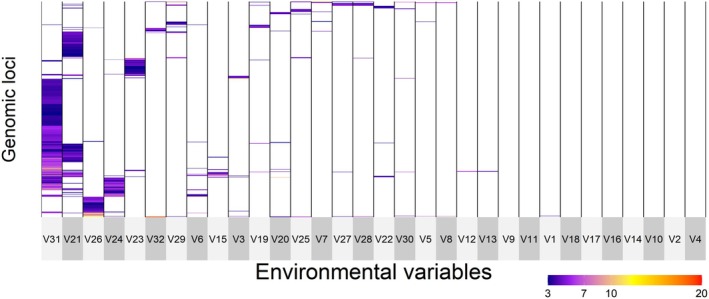
Covariation of 671 outlier single nucleotide polymorphisms (SNPs), as identified by gINLAnd, in the “all samples” data set with logBF values ≥ 3. The outlier SNPs are in rows with the associated environmental variables in columns. Environmental variables in columns correspond to V1:Depth of the seafloor (m), V2:East/West Aspect (radians), V3:North/South Aspect (radians), V4:Plan Curvature, V5:Profile Curvature, V6:Distance to Shore (km), V7:Bathymetric Slope (degrees), V8:Concavity (degrees), V9:Mean Annual Sea Surface Salinity (SSS) (psu), V10:SSS of the freshest month (psu), V11:SSS of the saltiest month (psu), V12:Annual range in SSS (psu), V13:Annual variance in SSS (psu), V14:Mean Annual Sea Surface Temperature (SST) (°C), V15:SST of the coldest month (°C), V16:SST of the warmest month (°C), V17:Annual range in SST (°C), V18:Annual variance in SST (°C), V19:Sst_trend_1982_2019: Number of days with SST > 26oC threshold, V20:Sst_trend_1982_2019: Number of days with SST < 12oC, V21:Sst_min1982_2019_slopes: Minimum annual SST, V22:Sst_mean1982_2019_slopes: Mean annual SST, V23:Sst_max1982_2019_slopes: Maximum annual SST, V24:Nitrate, V25:Bottom silicate, V26:Bottom phosphate, V27:Bottom Utilized Oxygen, V28:Bottom dissolved oxygen, V29:Bottom nitrate, V30:Seabed temperature, V31:PH, V32:Kinetic energy due to currents at the seabed in the Mediterranean Sea. For a full reference to the variables, see Table S2.

**FIGURE 4:**
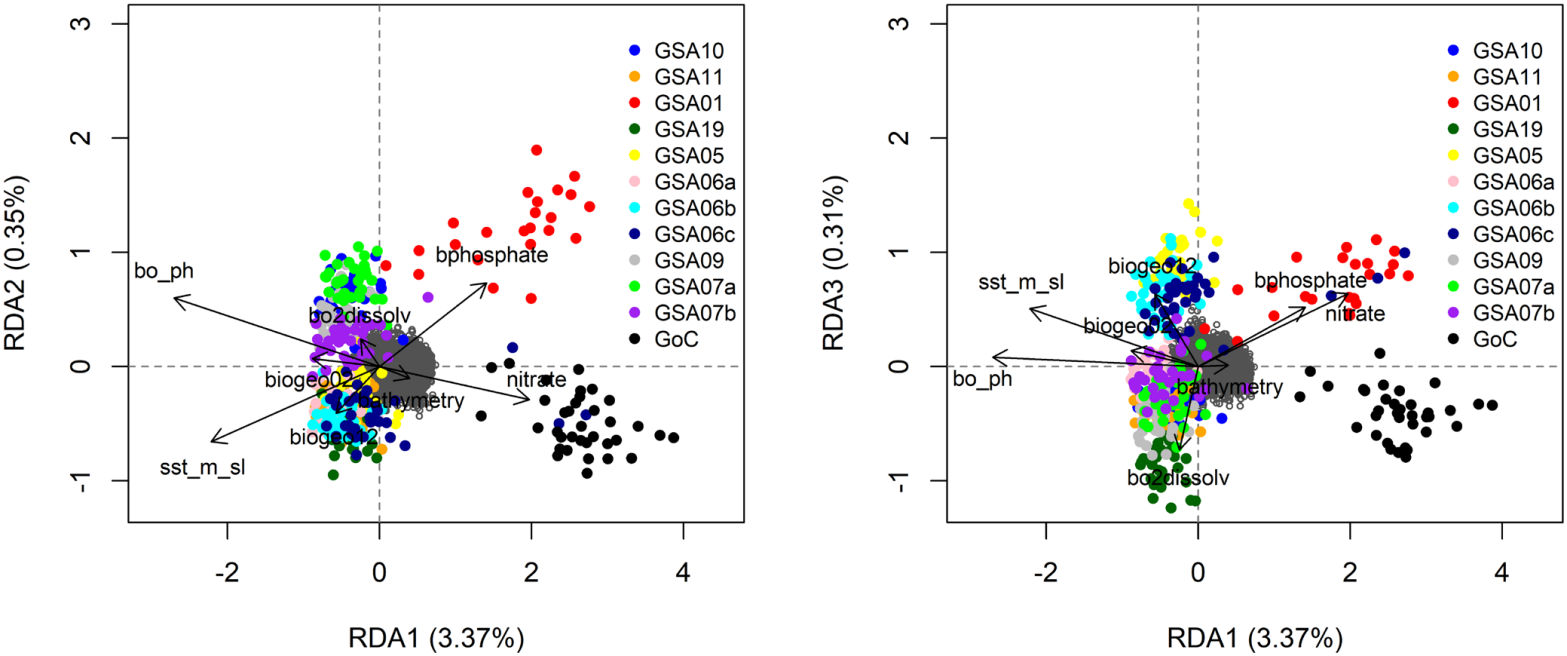
Triplots of the “all samples” data set for RDA axes 1 and 2 (left), and RDA axes 1 and 3 (right) for the “all samples” dataset. Single nucleotide polymorphisms (SNPs) are represented by the gray cloud of points at the center of the axes. The points represent anchovies with color coding by sampling site. Black vectors represent environmental predictors: bo_ph: PH, sst_m_sl:Sst_mean1982_2019_slopes: Mean annual SST, bathymetry: Depth of the seafloor (m), nitrate: Nitrate, bo2dissolv: Bottom dissolved oxygen, biogeo12:Annual variance in SSS (psu), bphosphate: Bottom phosphate, bogeo02:North/South Aspect (radians) (Table S2).

In the “all samples” dataset, gINLAnd detected 671 loci with logBF > 3, interpreted as having a statistical dependence with a certain environmental variable and therefore likely to belong to a genomic region under selection. These loci were associated with 28 environmental variables related to bathymetry, nutrients, temperature, topography, salinity, and pH. No genotype‐environment association was found for seven environmental variables (*biogeo01*, *biogeo03*, *biogeo09*, *biogeo13*, *biogeo15*, *biogeo16*, *biogeo17*, Table S2). Four SNP groups exhibited shared patterns of association across multiple environmental variables (Figure 3). gINLAnd indicated five environmental variables (related to temperature [*sst_min_sl*, *sst_max_sl*], nutrients [*bphosphate*, *nitrate*], and pH [*bo_ph*]) displaying high covariation with specific genomic regions (see Table S2 and Figure 3). The highest number of loci was invoked by two variables, namely *bo_ph* and *sst_min_sl* with 368 and 184 loci, respectively. All of the other environmental variables exhibited patterns of association with fewer SNPs (1 SNP—67 SNPs). Moreover, PCadapt detected 1422 outlier loci, with 392 of those also detected by gINLAnd (common outliers). After checking for uncorrelated variables with VIF < 10, the final RDA model (*p* = 0.001) was performed with eight environmental variables (*bathymetry*, *biogeo02*, *biogeo12*, *sst_m_sl*, *nitrate*, *bphosphate*, *bo2dissolv* and *bo_ph*), all being statistically significant while explaining only 5.3% of the observed genomic variance (adjusted *R* ^2^: 0.0334). *Sst_m_sl* (1.4%) and *nitrate* (1%) explained most of the variation while having correlation with 18 and 8 loci, respectively. 108 out of the 185 candidate SNPs were correlated with pH. There were four significant RDA axes, which returned 185 unique candidate loci (88 SNPs on RDA axis 1, 30 on RDA axis 2, 32 on RDA axis 3 and 39 on RDA axis 4), while showing shared patterns of association across several environmental variables. Out of 185 candidate outliers, 94 were also detected by gINLAnd. On the first RDA axis, a positive relationship was evident between individuals from the *ATL* group and a few from the GSA01 area with *nitrate* and *bphosphate* (Figure 4A). On the same axis, *bphosphate* was negatively correlated with GSA19, GSA06a, and GSA11 (Figure 4A,B). On the third RDA axis, a distinction of the GSA19 site from the *MED* group is observed (Figure 4B). Samples from GSA06b, GSA06c, and GSA05 appear distinct from those from GSA06a, GSA11, GSA07a, GSA07b, GSA09, and GSA10 on the RDA3 axis, with the first group being positively correlated with annual variance in SSS (biogeo12) and the second with bottom dissolved oxygen (bo2dissolv). Finally, 83 putative outlier SNPs were found in common between the three methods, 81 of which correlated with pH (*bo_ph*) and two with mean annual SST slopes (1982–2019, *sst_m_sl*).

**FIGURE 5:**
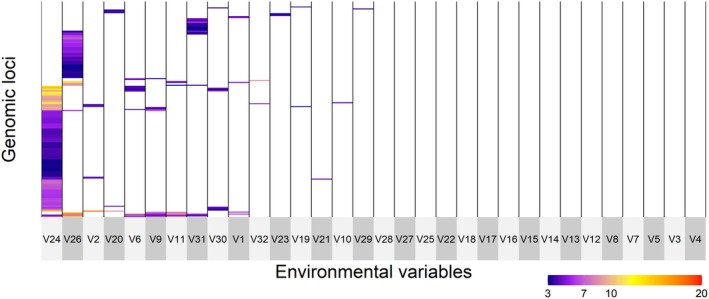
Covariation of 304 outlier single nucleotide polymorphisms (SNPs), as identified by gINLAnd, in the “MED” dataset with logBF values ≥ 3. The outlier SNPs are (in rows) with the associated and environmental variables (in columns). Environmental variables in columns correspond to V1:Depth of the seafloor (m), V2:East/West Aspect (radians), V3:North/South Aspect (radians), V4:Plan Curvature, V5:Profile Curvature, V6:Distance to Shore (km), V7:Bathymetric Slope (degrees), V8:Concavity (degrees), V9:Mean Annual Sea Surface Salinity (SSS) (psu), V10:SSS of the freshest month (psu), V11:SSS of the saltiest month (psu), V12:Annual range in SSS (psu), V13:Annual variance in SSS (psu), V14:Mean Annual Sea Surface Temperature (SST) (°C), V15:SST of the coldest month (°C), V16:SST of the warmest month (°C), V17:Annual range in SST (°C), V18:Annual variance in SST (°C), V19:Sst_trend_1982_2019: Number of days with SST > 26°C threshold, V20:Sst_trend_1982_2019: Number of days with SST < 12°C, V21:Sst_min1982_2019_slopes: Minimum annual SST, V22:Sst_mean1982_2019_slopes: Mean annual SST, V23:Sst_max1982_2019_slopes: Maximum annual SST, V24: Nitrate, V25: Bottom silicate, V26:Bottom phosphate, V27:Bottom Utilized Oxygen, V28:Bottom dissolved oxygen, V29:Bottom nitrate, V30:Seabed temperature, V31: PH, V32: Kinetic energy due to currents at the seabed in the Mediterranean Sea. For a full reference to the variables (see Table S2).

**FIGURE 6:**
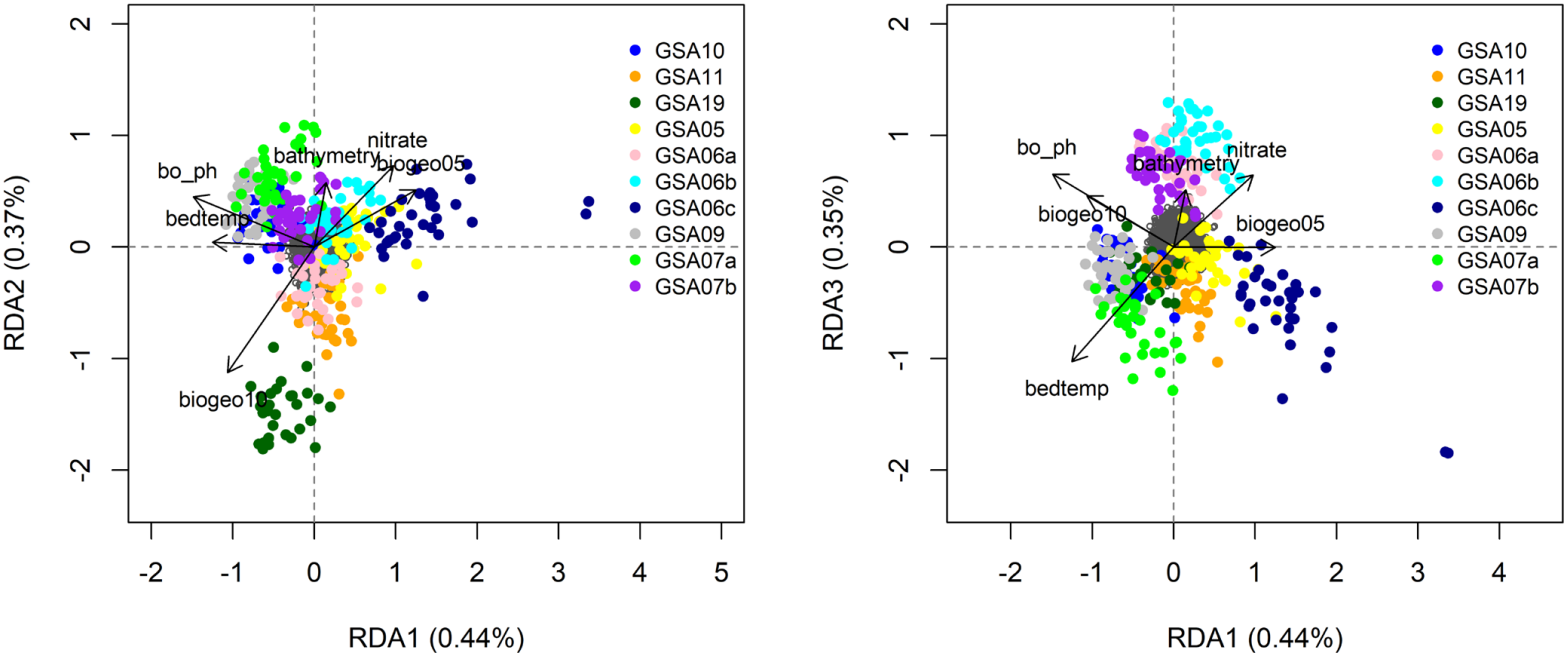
Triplots of the “all samples” data set for RDA axes 1 and 2 (left), and RDA axes 1 and 3 (right) for the *MED* dataset. Single nucleotide polymorphisms (SNPs) are represented by the gray cloud of points at the center of the axes. The points represent anchovies with color coding by sampling site. Black vectors represent environmental predictors: bo_ph: pH, bedtemp: Seabed temperature, bathymetry: Depth of the seafloor (m), nitrate: Nitrate, biogeo05: Distance to Shore (km), biogeo10: SSS of the saltiest month (psu) (Table S2).

In the *MED* dataset, gINLAnd detected 223 loci with logBF > 3, associated with 19 environmental variables related to bathymetry, nutrients, temperature, salinity, topography, and pH (Figure 5). Three SNP groups exhibited shared patterns of association across multiple environmental variables (Figure 5). Two environmental variables, that is, *nitrate* and *bphosphate* were found to be highly associated with 133 and 67 SNPs, respectively. Most of the other environmental variables exhibited patterns of association with fewer SNPs. PCadapt found 1181 outlier SNPs, 47 of which are in common with gINLAnd. The final RDA model was significant (*p* = 0.001), consisting of the same eight environmental variables as the “all samples” RDA model (see above), all being statistically significant, while explaining only 3.06% of the observed genomic variance (adjusted R‐squared: 0.003). All environmental variables explained an equal portion of the variation (29.8–35). Three RDA axes were significant, which identified 93 putative outlier loci (35 SNPs on RDA axis 1, 40 SNPs on RDA axis 2 and 18 on RDA axis 3). pH was associated with the greatest number of the candidate loci (33 SNPs) whereas distance to shore (*biogeo05*) was associated with the fewest number of loci (2 SNPs). A negative correlation between the GSA19 site with *nitrate* and *biogeo05* was observed on RDA axes 1 and 3 (Figure 6). Other positive relationships were those between GSA07a and GSA09 with seabed temperature (*bedtemp*, Figure 6).

**FIGURE 7:**
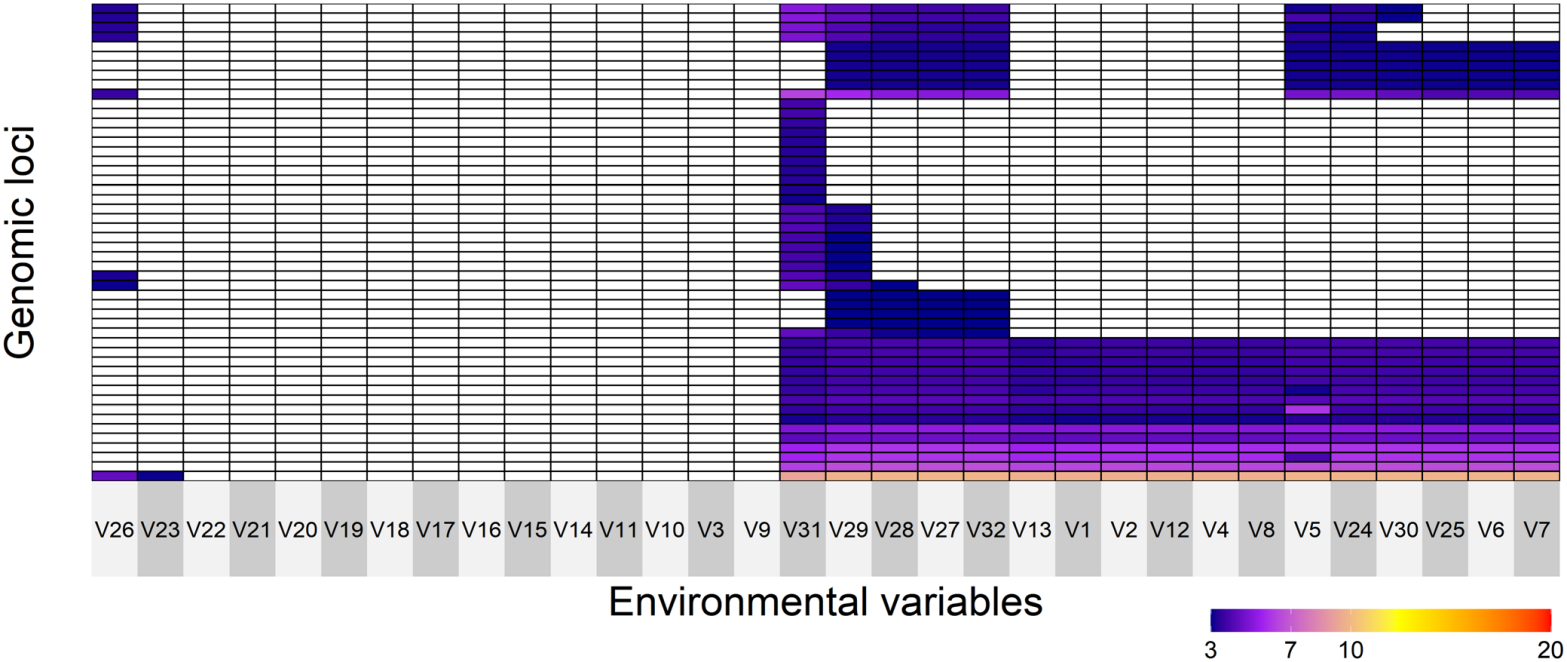
Covariation of 170 outlier single nucleotide polymorphisms (SNPs), as identified by gINLAnd, in the “ATL” dataset with logBF values ≥ 3. The outlier SNPs are (in rows) with the associated and environmental variables (in columns). Environmental variables in columns correspond to V1:Depth of the seafloor (m), V2:East/West Aspect (radians), V3:North/South Aspect (radians), V4:Plan Curvature, V5:Profile Curvature, V6:Distance to Shore (km), V7:Bathymetric Slope (degrees), V8:Concavity (degrees), V9:Mean Annual Sea Surface Salinity (SSS) (psu), V10:SSS of the freshest month (psu), V11:SSS of the saltiest month (psu), V12:Annual range in SSS (psu), V13:Annual variance in SSS (psu), V14:Mean Annual Sea Surface Temperature (SST) (°C), V15:SST of the coldest month (°C), V16:SST of the warmest month (°C), V17:Annual range in SST (°C), V18:Annual variance in SST (°C), V19:Sst_trend_1982_2019: Number of days with SST > 26°C threshold, V20:Sst_trend_1982_2019: Number of days with SST < 12°C, V21: Sst_min1982_2019_slopes: Minimum annual SST, V22:Sst_mean1982_2019_slopes: Mean annual SST, V23:Sst_max1982_2019_slopes: Maximum annual SST, V24: Nitrate, V25: Bottom silicate, V26:Bottom phosphate, V27:Bottom Utilized Oxygen, V28:Bottom dissolved oxygen, V29:Bottom nitrate, V30:Seabed temperature, V31: pH, V32: Kinetic energy due to currents at the seabed in the Mediterranean Sea. For a full reference to the variables (see Table S2).

In the *ATL* dataset, gINLAnd detected 170 loci with logBF > 3, associated with 15 environmental variables related to bathymetry, nutrients, temperature, salinity, topography and pH (Figure 7). Three SNP groups exhibited shared patterns of association across multiple environmental variables (Figure 5). Two environmental variables, *nitrate* and *bphosphate*, were highly associated with 110 and 50 SNPs, respectively. Most of the other environmental variables exhibited patterns of association with fewer SNPs. Finally, the RDA analysis was not possible since the two sites (GoC, GSA01) caused collinearity of all environmental variables.

Population genomic analyses on the dataset with SNPs that are putatively under selection (i.e., the 90 outlier SNPs identified in common by gINLAnd, PCadapt, and RDA) as well as on the neutral dataset (i.e., the 7748 SNPs that were not highlighted by any of the software in the “all samples” dataset) were consistent, both estimating *K* = 2 clusters. Individuals were allocated to the two clusters (the *MED* or the *ATL* cluster) in accordance with the results obtained when analyzing the SNP dataset containing all loci (Figures S1 and S2). The STRUCTURE bar plot based on the outlier SNPs only showed the same genetic pattern as the other datasets (all loci or neutral loci), but managed to clearly sort out some previously admixed samples to either cluster. For example, the GSA01, GoC and GSA06c have now fewer admixed individuals, i.e., 82%, 48% and 40%, respectively (Figure S2). DAPC density plots using the neutral markers found no admixed samples (the same with the dataset containing all loci), but that was not the case when the dataset with the outliers was used where admixed individuals were detected (Figure S4).

## 4 Discussion

Here, we used genome‐wide SNPs to investigate the population structure of the European anchovy at a fine spatial scale and incorporated environmental data to identify variables associated with the species' genetic variation. Our findings reveal a clear genetic differentiation between two major clusters, with the Almeria–Oran Front (AOF) acting as a biogeographic barrier. In addition, seascape features such as temperature, nutrient availability, salinity and dissolved oxygen appear to shape genetic variation across anchovy populations in the Western and Central Mediterranean Sea. Together, these results provide fruitful insights into the factors influencing anchovy population connectivity and contribute to a better understanding of the species' evolutionary and ecological dynamics.

### 4.1 Population Structure of Anchovies

There are multiple lines of evidence that support a marked population structure of *Engraulis encrasicolus* throughout its distribution (Ouazzani et al. 2017, and references therein). In this study, all clustering analyses (STRUCTURE, DAPC) and the genetic differentiation index (F_ST_), revealed a clear population structure of the European anchovy in the Western and Central Mediterranean Sea. In particular, both STRUCTURE and DAPC showed that the two‐cluster scenario best fits our genomic data. The *ATL* cluster consists of individuals from the GoC (Gulf of Cadiz) and the GSA01 (Alboran Sea) whereas the individuals from the remaining GSAs belong to the *MED* cluster. The observed pattern of genetic differentiation has also been reported in other studies (Bouchenak‐Khelladi et al. 2008; Zarraonaindia et al. 2012). These two clusters appear to be separated by the Almeria–Oran Front. In fact, multiple studies have reported the genetic distinctiveness of the Alboran anchovy population from the rest of the Mediterranean and, at the same time, its genetic similarity with the Cadiz population (Bembo et al. 1996; Bouchenak‐Khelladi et al. 2008; Magoulas et al. 2006; Sanz et al. 2008; Zarraonaindia et al. 2012; Ouazzani et al. 2016, but see also Viñas et al. 2013).

The biogeographic boundary created by the AOF may not completely prevent gene flow among anchovy populations. The AOF is adjacent to the GSA01 (Alboran Sea) and GSA06c (Southeastern Spain), where many individuals showed mixed ancestry. All individuals from the GSA01 and 19 from GSA06c (i.e., 54%) were admixed. Zarraonaindia et al. (2012) also found admixed individuals in the Alboran Sea. Therefore, the identification of admixed individuals in the Gulf of Cadiz and other Mediterranean sites is expected given their connection to the Alboran and GSA06c (southeastern Spain), respectively.

Although anchovies show strong structure at broader scales, no genetic differentiation was detected within the Western Mediterranean populations (except the GSA01, e.g., Sanz et al. 2008). This region is characterized by specific water circulation patterns, i.e., marine currents, promoting the passive egg and larvae transport from the spawning areas and hence the mixing of individuals from remote regions (Catanese et al. 2020). In particular, on the western coasts of the Italian peninsula, the Tyrrhenian and Ligurian seas are connected via the Corsica Channel, where the “Ligurian Current” begins (Astraldi et al. 1995) and flows along the continental slope, at least as far as the Channel of Ibiza (Millot 1999). Astraldi et al. (1994) and Viettia et al. (2010) have reported westward movements of European anchovy's eggs and larvae by coastal currents in the Tyrrhenian and Ligurian seas. Also, Rubín (1996) reported a mechanism of egg and larval transport from the Gulf of Cadiz to the Alboran Sea via the inflow of the Atlantic jet. Therefore, anchovy eggs and larvae could passively migrate, taking advantage of such currents, eventually mixing with the indigenous anchovy's early life stages. As they grow into adults, they reproduce locally, generating the observed genetic pattern suggesting panmixia in the considered area.

Another important biogeographic boundary within the Mediterranean Sea is the Siculo‐Tunisian Strait (STS; Zardoya et al. 2004), where two opposing currents meet: the eastward Atlantic‐Ionian Stream (AIS, Robinson et al. 1999) and the westward Ionian current. Such hydrographic patterns bound the natural dispersal across the STS for several fish species, such as *Pomatoschistus tortonesei* (Mejri et al. 2009), *Pomatoschistus minutus* (Stefanni and Thorley 2003), and *Scomber scombrus* (Zardoya et al. 2004). Ruggeri et al. (2016) found high genetic similarity between the southern Adriatic GSA17 and GSA18 (Bari and Pescara regions), the Northwestern Sicily GSA10 (Castellammare del Golfo region) and the Western Italy GSA10 (Sperlonga region), although little is known about the genetic relationship among the GSA19, GSA10, GSA15 and GSA16 (Bembo et al. 1996; Borsa 2002; Cuttitta et al. 2015). Here, the GSA19 (Western Ionian Sea) was found to be genetically homogeneous to the GSA10 (Tyrrhenian Sea) and GSA09 (Ligurian Sea) in all tests performed (Figure 2A and Figure S3). Thus, the genetic similarity implies potential high connectivity. Several studies have described the eastward transport of anchovy eggs and larvae through the Sicily Channel (STS) (e.g., Bonanno et al. 2013; Falcini et al. 2015; García Lafuente et al. 2002). Falcini et al. (2015) indicated the role of wind‐induced coastal currents in influencing the fate and distribution of European anchovy larvae within the Sicily Channel. The AIS controls the surface circulation of the Sicilian Channel and has a key role in transferring the anchovy's eggs and larvae eastward (García Lafuente et al. 2002). Thus, the particular oceanographic features of the STS likely facilitate genetic connectivity between populations on either side of Sicily.

Pairwise F_ST_ values (Table S3) support the clustering analyses (STRUCTURE and DAPC). For instance, when comparing the Gulf of Cadiz population with ones from the *MED* cluster, a substantial F_ST_ (> 0.085) is observed. Pairwise F_ST_ values ranged from 0.026 to 0.128 (statistically significant comparisons only). Low *F*_ST_ values were found in the *MED* cluster and high ones when comparing GSAs from the two clusters. Notably, the two clusters display a significant pairwise *F*_ST_ of 0.097. Our *F*_ST_ measurements are in alignment with other studies on the species under study: Catanese et al. (2020) used a 96 SNP‐panel for the Western Italy anchovy populations and found *F*_ST_ values from 0.011 to 0.129, and lastly, Zarraonaindia et al. (2012) using 90 SNPs (nuclear and mitochondrial) found a range of *F*_ST_ values (0.04–0.318) from Denmark to South Africa (average *F*_ST_ = 0.078) (see also Huret et al. 2020). Finally, such high *F*_ST_ values contrast with the generally low differentiation observed in most marine fish species (Magoulas et al. 2006; Ward et al. 1994) Because our estimates are based on a genome‐wide SNP dataset, they not only offer comparable results but also highlight the observed genetic differentiation among specific anchovy populations.

### 4.2 How the Environment Impacts the European Anchovy

Temperature and food availability changes may significantly affect the abundance of anchovy populations (Schwartzlose et al. 1999). Temperature influences the total length (LT) (Pauly 1980; Basilone et al. 2004) and the timing of spawning onset (Furnestin and Furnestin 1959). For the European anchovy, the minimum spawning temperature is approximately 13°C (Fage 1920), with spawning intensifying as the temperature rises (Palomera 1992; Palomera et al. 2007). The processes of migration from the coast towards deeper waters at the beginning of the reproductive season are temperature‐dependent mechanisms (Alheit et al. 2012; Mutalipassi et al. 2024). In our study and considering the “all samples” dataset, 369 and 18 SNPs (detected by gINLAnd and RDA, respectively) were identified to have a high association with temperature‐related variables. In fact, two candidate SNPs found in common by gINLAnd and RDA were associated with *sst_m_sl* (slopes of temporal linear regression for the mean annual SST). Moreover, minimum and maximum annual SST were found to represent high covariation with specific genomic regions consisting of many candidate outlier SNPs (Figures 3 and 5). On the other hand, temperature could act as a proxy for upwelling conditions (Tapia et al. 2009) favoring anchovy growth and reproduction (Basilone et al. 2020).

The European anchovy as “income breeder” (sensu Stearns 1992; Albo‐Puigserver et al. 2020) relies on food intake during the reproductive period to meet its energy demands, making food availability crucial for reproductive success. Egg production (Somarakis et al. 2004), mortality rate at the embryonic stage (Kim and Lo 2001) and recruitment (Borja et al. 1998) are influenced by food availability. In our study, 197 and 24 SNPs were detected by gINLAnd and RDA, respectively, to have high association with nutrient‐related variables (*nitrate*, *phosphate*) in the “all samples” dataset. Two different genomic clusters were found to have high covariation with nitrate and bottom phosphate (Figures 3 and 5). Finally, the significance of nutrient‐related variables is observed in the *MED* and *ATL* groups also, since most of the outlier SNPs have high covariation with nitrate, phosphate and silicate (Figures 3, 5 and 7).

Dissolved oxygen and salinity were also found to be associated with genomic variation in anchovy populations from the western and central Mediterranean Sea. Regarding dissolved oxygen (*bo2dissolv*), it plays a crucial role in anchovy spawning time and early larval development (Ruggeri et al. 2016). Our study shows that dissolved oxygen concentration is associated with specific polymorphisms (Figure 3) and is correlated with specific GSAs, for example, GSA09 (Figure 5). In anchovies, such associations have also been reported by Ruggeri et al. (2016), who analyzed samples from either side of the Italian peninsula. As for salinity, our results indicate that its annual variance in sea surface salinity (*biogeo12*) has a modest effect on anchovy genetic variation, particularly in the *MED* dataset (Figures 4 and 6). In the *ATL* dataset, only a few, statistically significant SNPs were found to covary with *biogeo12* (Figure 7). Salinity has been previously used to explain anchovy variability in the Mediterranean, reinforcing its importance in anchovy studies (e.g., Pennino et al. 2020 in the NW Mediterranean Sea; Džoić et al. 2022 in the Adriatic Sea).

Environmental variables such as temperature, salinity, oxygen, and nutrients can impose selective pressures that shape genomic variation in small pelagic fishes, including anchovy. Temperature gradients may favor allelic variation in genes linked to energy metabolism and thermal stress tolerance (Somero 2012). Variation in oxygen availability can select on hypoxia‐response pathways, including components of mitochondrial respiration (e.g., cytochrome c oxidase subunits), which modulate aerobic capacity and cellular energetics (Bremer 2022). Differences in salinity can drive selection on osmoregulatory machinery that maintains ion balance and water flux across gill epithelia (Tine et al. 2014). In anchovy specifically, previous work has highlighted differentiation at genes related to circadian regulation and reproduction, consistent with adaptation to spatial variation in environmental regimes and spawning conditions (Montes et al. 2016; Pujolar et al. 2025; Bonhomme et al. 2022). Together, these candidate pathways provide plausible mechanisms by which temperature, oxygen, and salinity regimes could shape allele‐frequency differences among regions. While our study does not test locus‐specific associations, these hypotheses offer a framework for future environmental association and functional analyses in anchovy populations. Nutrient availability, particularly nitrate and phosphate, can also influence anchovy adaptation by shaping primary productivity and food‐web dynamics (Silva et al. 2014). Variability in these nutrients affects the abundance and quality of zooplankton prey, thereby indirectly imposing selective pressures on metabolic and reproductive pathways. In this context, genes involved in oxidative phosphorylation (OXPHOS) are particularly relevant, as efficient ATP production is critical for meeting the high energetic demands of small pelagic fishes (Saraste 1999; Baltazar‐Soares et al. 2021). Signals of diversifying selection in OXPHOS genes, as observed in other pelagic species (Pappalardo et al. 2024; Calogero et al. 2025; Sebastian et al. 2020), highlight their potential role in mediating adaptive responses to fluctuating nutrient regimes in anchovy populations. Nevertheless, despite these plausible mechanisms, our genome‐wide analysis did not reveal fine‐scale genetic differentiation that could explain the regional differences in anchovy's biological traits reported for Mediterranean populations (e.g., Brosset et al. 2017; Van Beveren et al. 2016). Such results are consistent with evidence that phenotypic variability in fishes can be high and largely independent of genetic divergence (Ihssen et al. 1981; Ryman et al. 1984). Similar patterns have been observed in *Engraulis mordax* , which exhibits high morphological variation independent of genetic or geographical structuring (Spanakis et al. 1989; Nelson et al. 1994). This suggests that the observed regional variability in anchovy is more likely driven by environmentally induced plasticity rather than by underlying genetic differentiation.

### 4.3 Management Implications

Our study provides crucial insights for the effective management of the European anchovy fisheries by identifying distinct genetic populations and the environmental variables influencing their distribution. Recognizing the genetic differentiation between the Alboran and northwestern Mediterranean populations supports the need for region‐specific management strategies. Mismanagement, such as treating genetically distinct populations as a single unit (type II error; Waples et al. 2008), could lead to stock collapse due to overfishing and loss of genetic variation in key adaptive genes, reducing the species' capacity to respond to environmental changes (Viñas et al. 2011; Marty et al. 2015). Although genetic connectivity exists in the northwestern and central Mediterranean (*MED* group) due to passive egg and larval transport via marine currents, effective management requires the identification and protection of each population and spawning ground through fishing activity limitations (Roberts et al. 2005). Without sustainable management, commercial fishery collapse – and even species extinction‐ could become inevitable (Hutchings 2001). Therefore, implementing genetic monitoring of anchovy stocks is vital to safeguard *Engraulis encrasicolus* as both a biological species and a valuable commercial resource (Sanz et al. 2008).

## Author Contributions

**Damianos Alexandridis:** formal analysis (lead), investigation (lead), methodology (equal), software (lead), writing – original draft (lead), writing – review and editing (lead). **Tereza Manousaki:** conceptualization (equal), data curation (equal), formal analysis (equal), funding acquisition (equal), investigation (equal), methodology (equal), resources (equal), software (equal), supervision (equal), writing – original draft (equal), writing – review and editing (equal). **Aglaia Antoniou:** data curation (equal), funding acquisition (equal), investigation (supporting), methodology (supporting), software (supporting), supervision (equal), writing – review and editing (supporting). **Jon Kristoffersen:** investigation (supporting), writing – review and editing (supporting). **Charis Apostolidis:** resources (supporting). **Rita Cannas:** resources (supporting), writing – review and editing (supporting). **Maria Teresa Spedicato:** funding acquisition (equal), resources (supporting), writing – review and editing (supporting). **Alessia Cariani:** writing – review and editing (supporting). **Jose María Bellido:** writing – review and editing (supporting). **Antonios Magoulas:** writing – review and editing (supporting). **Francisco Ramírez:** writing – review and editing (supporting). **Elena Lloret‐Lloret:** writing – review and editing (supporting). **Marta Albo‐Puigserver:** writing – review and editing (supporting). **Marta Coll:** funding acquisition (lead), investigation (equal), project administration (lead), resources (lead), supervision (equal), writing – original draft (equal), writing – review and editing (supporting). **Costas S. Tsigenopoulos:** conceptualization (equal), funding acquisition (equal), methodology (equal), supervision (equal), writing – original draft (equal), writing – review and editing (supporting).

## Conflicts of Interest

The authors declare no conflicts of interest.

## Supporting information


**Figure S1:** DAPC densi plot using neutral SNPs.

**Figure S2:** DAPC densi plot using outlier SNPs.

**Figure S3:** DAPC MED group.

**Figure S4:** STRUCTURE bar plot across all sampling sites using neutral SNPs.

**Figure S5:** STRUCTURE bar plot across all sampling sites using outlier SNPs.

**Figure S6:** Outlier detection by three methods for the “all samples” dataset.

**Table S1:** Sampling (GSA Geographical Sub‐Areas of the Mediterranean where samples were collected from; M male; F female; i immature; na = not available) and sequencing details.

**Table S2:** Relevant raw and derived abiotic and biotic variables (i.e., physical, chemical and biological variables) that were employed in the tests of genotype–environmental correlation and their implications on the habitat and biology of the species, though not exclusive.

**Table S3:** Pairwise Fst values with significance level of 0.05 and 10,000 permutations among the studied areas. Statistically significant values are in bold.

**Data S1:** Supporting Information.

## Data Availability

Raw reads and the generated genome assembly have been deposited in the European Nucleotide Archive (ENA) at EMBL‐EBI under accession numbers PRJEB88383 (https://www.ebi.ac.uk/ena/browser/view/PRJEB88383) and GCA_976921385 (https://www.ebi.ac.uk/ena/browser/view/GCA_976921385), respectively. A VCF file with all filtered and unlinked SNPs used in the analyses of this study is provided as an additional file.
