# Supplementary figures and images for: Uncovering the Genetic Structure of European Anchovy Populations in Central and Western Mediterranean
Source: Ecol Evol. 2025 Nov 18;15(11):e72441. doi: 10.1002/ece3.72441 (PMC12626725; doi:10.1002/ece3.72441)

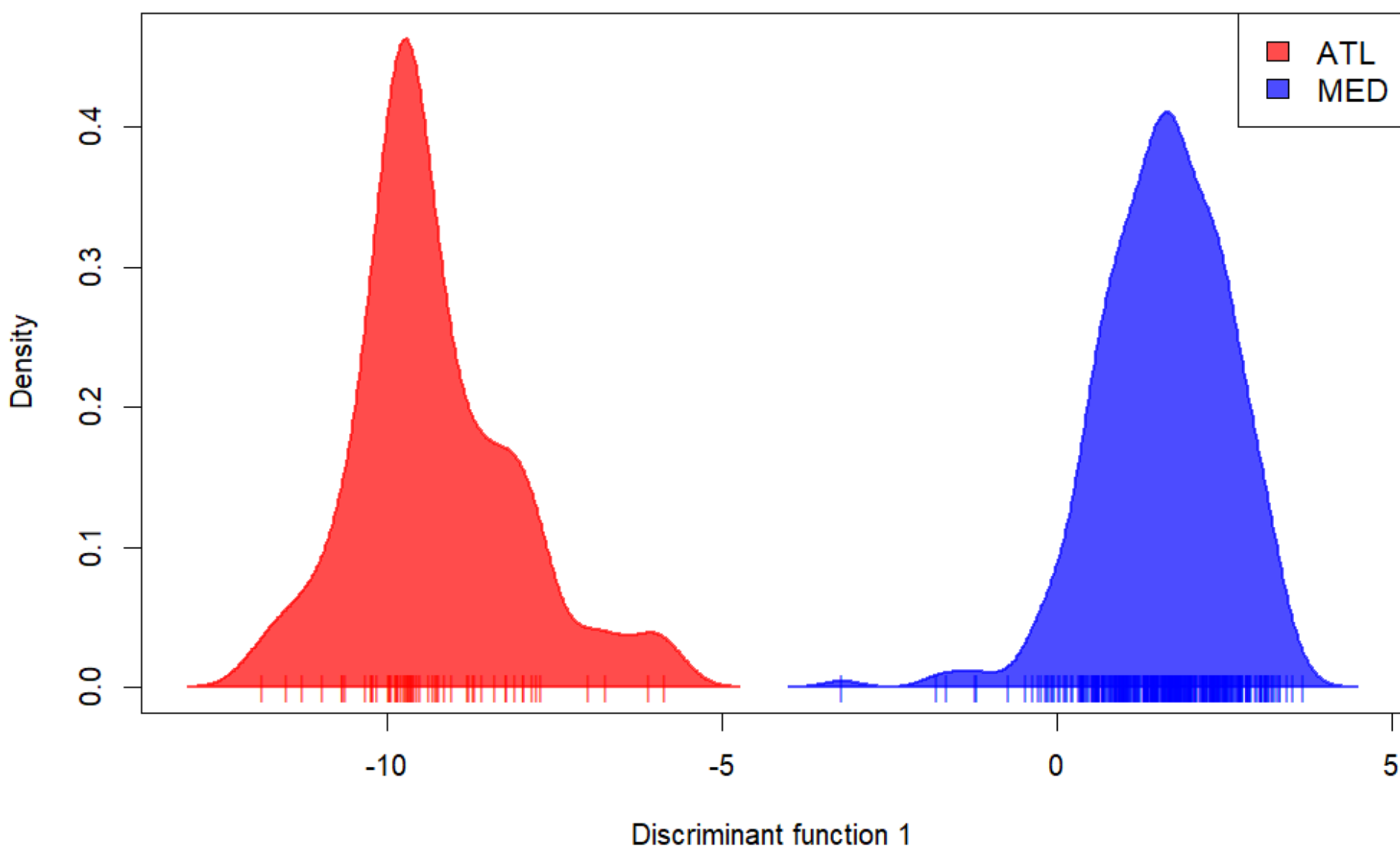

Figure S1: DAPC density plot using neutral SNPs.

Supplement: Supplementary file 1 — Figure S1: DAPC densi plot using neutral SNPs. [file ECE3-15-e72441-s008.pdf]

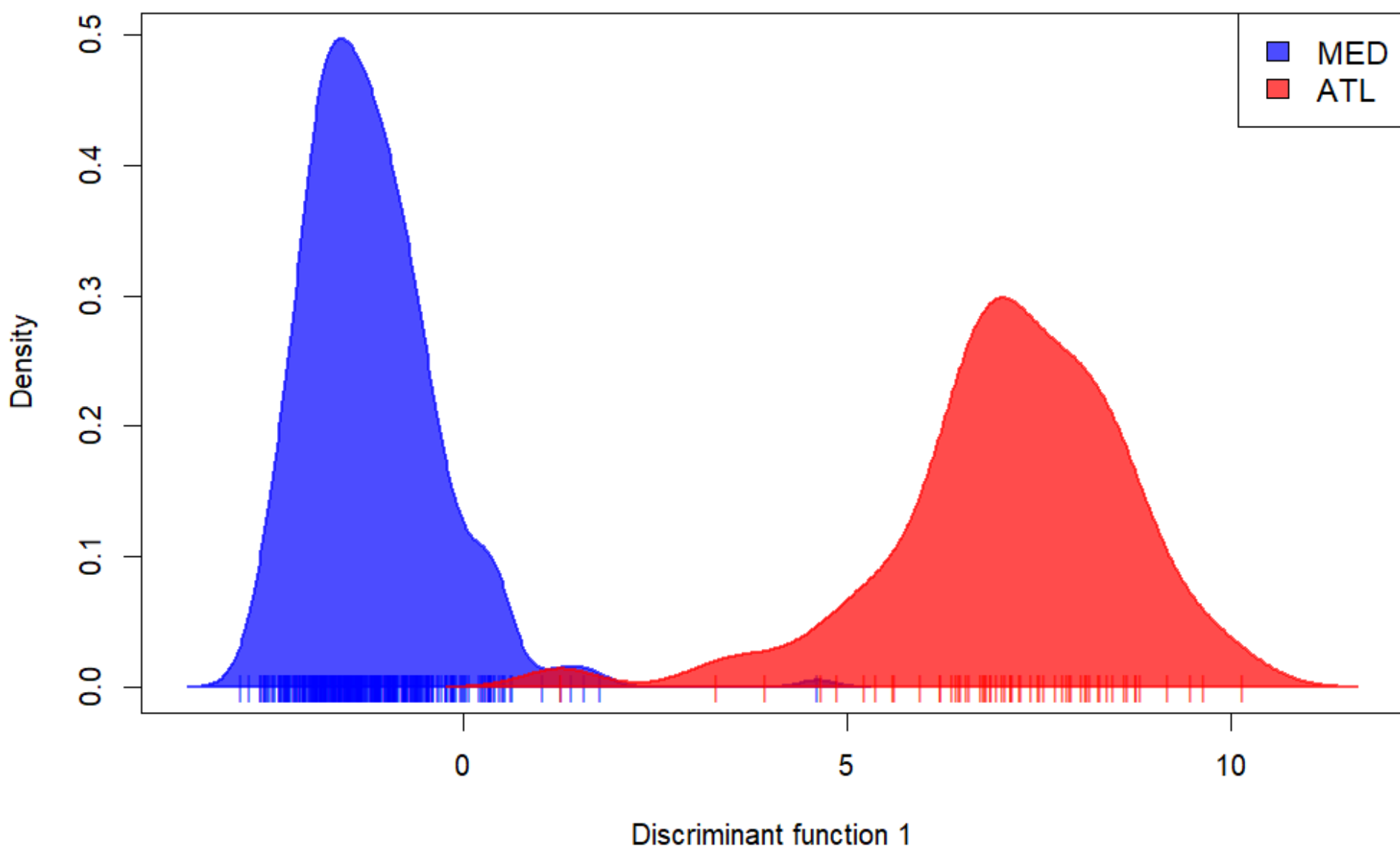

Figure S2: DAPC density plot using outlier SNPs.

Supplement: Supplementary file 2 — Figure S2: DAPC densi plot using outlier SNPs. [file ECE3-15-e72441-s001.pdf]

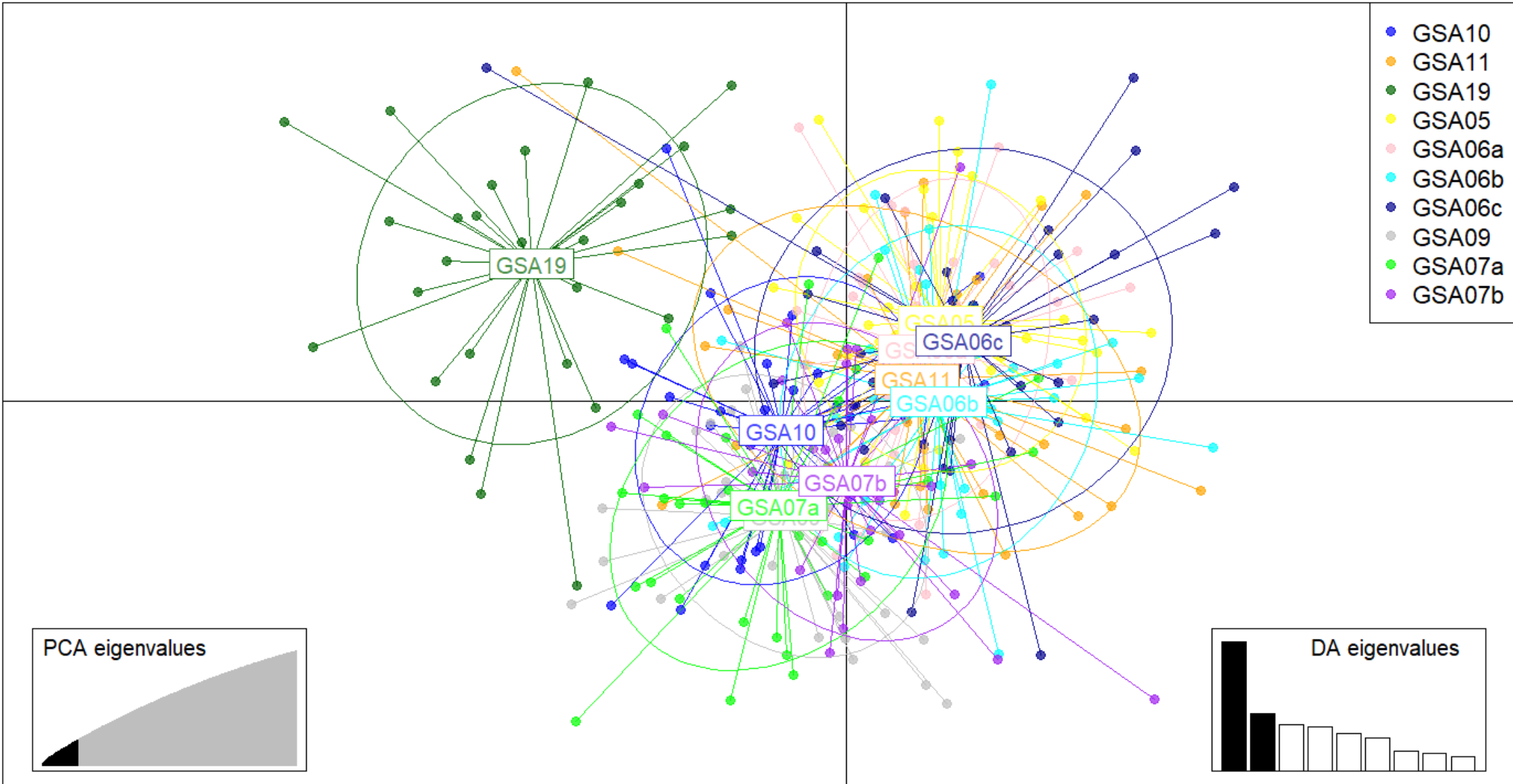

Figure S3: DAPC MED group.

Supplement: Supplementary file 3 — Figure S3: DAPC MED group. [file ECE3-15-e72441-s003.pdf]

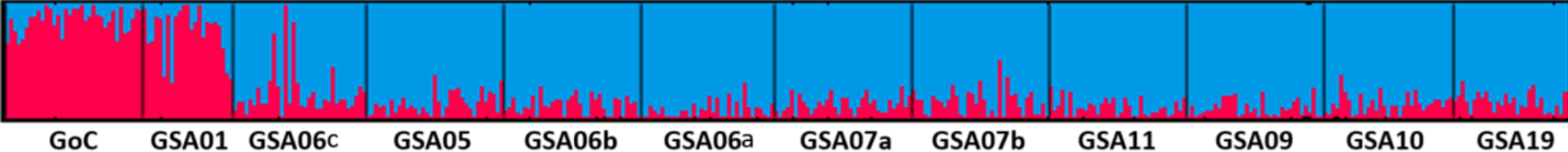

Figure S4: STRUCTURE bar plot across all sampling sites using neutral SNPs.

Supplement: Supplementary file 4 — Figure S4: STRUCTURE bar plot across all sampling sites using neutral SNPs. [file ECE3-15-e72441-s002.pdf]

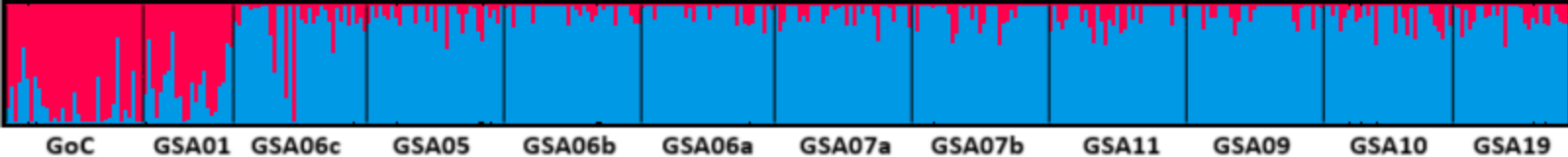

Figure S5: STRUCTURE bar plot across all sampling sites using outlier SNPs.

Supplement: Supplementary file 5 — Figure S5: STRUCTURE bar plot across all sampling sites using outlier SNPs. [file ECE3-15-e72441-s006.pdf]

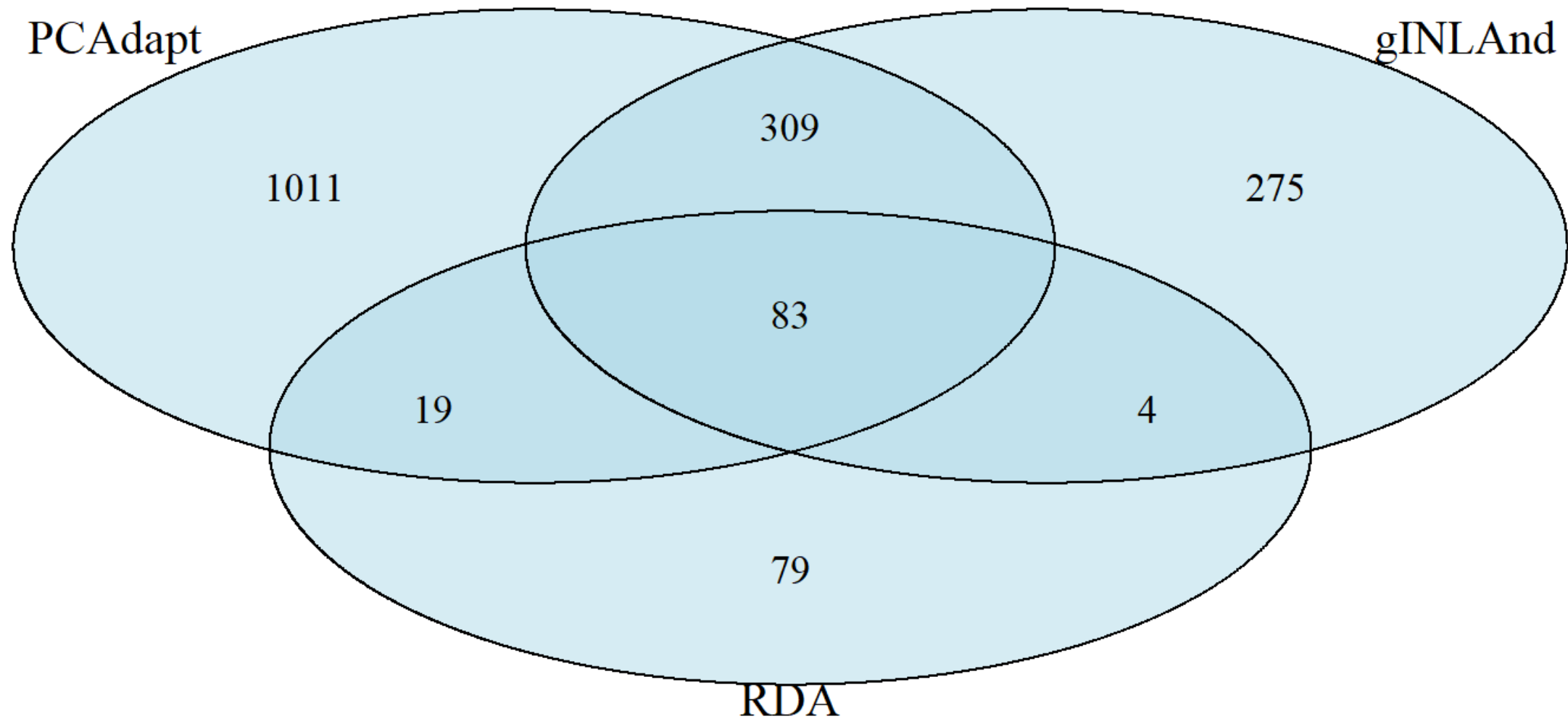

Figure S6: Outlier detection by three methods for the “all samples” dataset.

Supplement: Supplementary file 6 — Figure S6: Outlier detection by three methods for the “all samples” dataset. [file ECE3-15-e72441-s010.pdf]
