# Supplementary material for: Uncovering the Genetic Structure of European Anchovy Populations in Central and Western Mediterranean
Source: Ecol Evol. 2025 Nov 18;15(11):e72441. doi: 10.1002/ece3.72441 (PMC12626725; doi:10.1002/ece3.72441)
Supplement: Supplementary file 7 — Table S1: Sampling (GSA Geographical Sub‐Areas of the Mediterranean where samples were collected from; M male; F female; i immature; na = not available) and sequencing details. [file ECE3-15-e72441-s004.pdf]

Table S1: Sampling (GSA Geographical Sub-Areas of the Mediterranean where samples were collected from; M male; F female; i immature; na= not available) and sequencing details.

**GSA**	**Location**	**Location code**	**Latitude**	**Longitude**	**Mean depth (m)**	**Number of samples**	**Sex (F:M:i:NA)**	**Mean number of retained reads (%)**
**ATL**	Gulf of Cadiz	GoC	36.548500°	-6.567900°	70	35	18:17	2,323,766 (97.12%)
**1**	Malaga	GSA01	36.322800°	-4.304600°	50	24	13:11	1,927,015 (97.8%)
**6**	Rosas	GSA06a	42.197311°	3.171022°	32	35	15:20	1,849,811 (98.46%)
**6**	Tarragona	GSA06b	41.055556°	1.345833°	88	35	18:10:7	2,588,248 (98.47%)
**6**	Torrevieja	GSA06c	38.014400°	-0.243100°	62	35	22:12:1	2,511,458 (98.1%)
**5**	Mallorca	GSA05	39.484394°	2.616792°	44	35	21:12:2	2,123,592 (98.29%)
**7**	Gulf Lions	GSA07a	43.306944°	3.636111°	30	35	14:4:8:9	2,596,354 (97.49%)
**7**	Gulf Lions	GSA07b	42.498786°	3.392842°	111	35	16:18:1	1,689,671 (98.09%)
**9**	Elba	GSA09	42.890283°	10.428333°	70	35	21:14	2,159,477 (97.44%)
**10**	Torre Annunziata	GSA10	40.668800°	14.392500°	90	35	5:30	2,345,234 (96.6%)
**11**	Buggerru	GSA11	39.42100°	8.369667°	45	35	11:21:3	2,863,847 (97.65%)
**19**	Cirò Marina	GSA19	39.402700°	17.146200°	30	34	12:22	3,124,525 (96.19%)
