# Supplementary material for: Uncovering the Genetic Structure of European Anchovy Populations in Central and Western Mediterranean
Source: Ecol Evol. 2025 Nov 18;15(11):e72441. doi: 10.1002/ece3.72441 (PMC12626725; doi:10.1002/ece3.72441)
Supplement: Supplementary file 8 — Table S2: Relevant raw and derived abiotic and biotic variables (i.e., physical, chemical and biological variables) that were employed in the tests of genotype–environmental correlation and their implications on the habitat and biology of the species, though not exclusive. [file ECE3-15-e72441-s007.pdf]

Table S2. Relevant raw and derived abiotic and biotic variables (i.e., physical, chemical and biological variables) that were employed in the tests of genotype–environmental correlation and their implications on the habitat and biology of the species, though not exclusive.

a/a	*Variable_Name*	*Description*	*Categories*	*Source*
1	bathymetry	Depth of the seafloor (m)	topography	MARSPEC
2	biogeo01	East/West Aspect (radians)	topography	MARSPEC
3	biogeo02	North/South Aspect (radians)	topography	MARSPEC
4	biogeo03	Plan Curvature	topography	MARSPEC
5	biogeo04	Profile Curvature	topography	MARSPEC
6	biogeo05	Distance to Shore (km)	topography	MARSPEC
7	biogeo06	Bathymetric Slope (degrees)	topography	MARSPEC
8	biogeo07	Concavity (degrees)	topography	MARSPEC
9	biogeo08	Mean Annual Sea Surface Salinity (SSS) (psu)	salinity	MARSPEC
10	biogeo09	SSS of the freshest month (psu)	salinity	MARSPEC
11	biogeo10	SSS of the saltiest month (psu)	salinity	MARSPEC
12	biogeo11	Annual range in SSS (psu)	salinity	MARSPEC
13	biogeo12	Annual variance in SSS (psu)	salinity	MARSPEC
14	biogeo13	Mean Annual Sea Surface Temperature (SST) (°C)	temperature	MARSPEC
15	biogeo14	SST of the coldest month (°C)	temperature	MARSPEC
16	biogeo15	SST of the warmest month (°C)	temperature	MARSPEC
17	biogeo16	Annual range in SST (°C)	temperature	MARSPEC
18	biogeo17	Annual variance in SST (°C)	temperature	MARSPEC
19	sst_tr26	sst_trend_1982_2019: number of days with SST >26oC threshold	temperature	Ramirez et al. 2018; 2021
20	sst_tr12	sst_trend_1982_2019: number of days with SST <12oC	temperature	Ramirez et al. 2018; 2021
21	sst_min_sl	sst_min1982_2019_slopes: minimum annual SST	temperature	Ramirez et al. 2018; 2021
22	sst_m_sl	sst_mean1982_2019_slopes: mean annual SST	temperature	Ramirez et al. 2018; 2021
23	sst_max_sl	sst_max1982_2019_slopes: maximum annual SST	temperature	Ramirez et al. 2018; 2021
24	nitrate	nitrate	nutrients	GMED
25	bsilicate	bottom silicate	nutrients	GMED
26	bphosphate	Bottom phosphate	nutrients	GMED
27	bo2utilize	Bottom Utilized Oxygen	nutrients	GMED
28	bo2dissolv	Bottom dissolved oxygen	nutrients	GMED

29	bnitrate	bottom nitrate	nutrients	GMED
30	bedtemp	Seabed temperature	temperature	GMED
31	bo_ph	pH	chemicals	GMED
32	Currents	Kinetic energy due to currents at the seabed in the Mediterranean Sea	climatology	EMODNET
