# Supplementary material for: Uncovering the Genetic Structure of European Anchovy Populations in Central and Western Mediterranean
Source: Ecol Evol. 2025 Nov 18;15(11):e72441. doi: 10.1002/ece3.72441 (PMC12626725; doi:10.1002/ece3.72441)
Supplement: Supplementary file 9 — Table S3: Pairwise Fst values with significance level of 0.05 and 10,000 permutations among the studied areas. Statistically significant values are in bold. [file ECE3-15-e72441-s009.pdf]

Table S3: Pairwise Fst values with significance level of 0.05 and 10,000 permutations among the studied areas. Statistically significant values are in bold.

**Area**	**GoC**	**GSA01**	**GSA06c**	**GSA05**	**GSA06b**	**GSA06c**	**GSA07a**	**GSA07b**	**GSA11**	**GSA09**	**GSA10**
**GoC**											
**GSA01**	0.0043										
**GSA06c**	**0.0851**	**0.0504**									
**GSA05**	**0.1077**	**0.0689**	0.0009								
**GSA06 b**	**0.1190**	**0.0774**	0.0028	0.0019							
**GSA06a**	**0.1277**	**0.0867**	**0.0069**	**0.0026**	0.0009						
**GSA07a**	**0.1090**	**0.0725**	0.0014	0.0017	0.0028	0.0017					
**GSA07 b**	**0.1136**	**0.0752**	0.0006	0.0021	0.0027	0.0021	0.023				
**GSA11**	**0.1166**	**0.0751**	**0.0041**	0.002	0.0018	0.0014	0.0127	0.0016			
**GSA09**	**0.1232**	**0.0807**	0.0009	0.0016	0.0035	0.0026	0.0018	0.0014	0.0025		
**GSA10**	**0.1081**	**0.0732**	0.0024	0.0041	0.0042	0.009	0.0011	0.0012	0.0035	0.0021	
**GSA19**	**0.1143**	**0.0769**	**0.0057**	**0.0033**	**0.0039**	**0.0040**	0.0023	**0.0029**	0.0039	0.0024	0.0001
